# A metabolic synthetic lethal strategy with arginine deprivation and chloroquine leads to cell death in ASS1-deficient sarcomas

**DOI:** 10.1038/cddis.2016.232

**Published:** 2016-10-13

**Authors:** Gregory R Bean, Jeff C Kremer, Bethany C Prudner, Aaron D Schenone, Juo-Chin Yao, Matthew B Schultze, David Y Chen, Munir R Tanas, Douglas R Adkins, John Bomalaski, Brian P Rubin, Loren S Michel, Brian A Van Tine

**Affiliations:** 1 Division of Medical Oncology, Department of Internal Medicine, Washington University in St. Louis School of Medicine, St. Louis, MO, USA; 2 Anatomic Pathology, Cleveland Clinic, Cleveland, OH, USA; 3 Siteman Cancer Center, Washington University in St. Louis School of Medicine, St. Louis, MO, USA; 4 Polaris Group, San Diego, CA, USA

## Abstract

Sarcomas comprise a large heterogeneous group of mesenchymal cancers with limited therapeutic options. When treated with standard cytotoxic chemotherapies, many sarcomas fail to respond completely and rapidly become treatment resistant. A major problem in the investigation and treatment of sarcomas is the fact that no single gene mutation or alteration has been identified among the diverse histologic subtypes. We searched for therapeutically druggable targets that are common to a wide range of histologies and hence could provide alternatives to the conventional chemotherapy. Seven hundred samples comprising 45 separate histologies were examined. We found that almost 90% were arginine auxotrophs, as the expression of argininosuccinate synthetase 1 was lost or significantly reduced. Arginine auxotrophy confers sensitivity to arginine deprivation, leading temporarily to starvation and ultimately to cell survival or death under different circumstances. We showed that, in sarcoma, arginine deprivation therapy with pegylated arginine deiminase (ADI-PEG20) maintains a prolonged state of arginine starvation without causing cell death. However, when starvation was simultaneously prolonged by ADI-PEG20 while inhibited by the clinically available drug chloroquine, sarcoma cells died via necroptosis and apoptosis. These results have revealed a novel metabolic vulnerability in sarcomas and provided the basis for a well-tolerated alternative treatment strategy, potentially applicable to up to 90% of the tumors, regardless of histology.

Sarcomas are a highly heterogeneous group of diseases comprising over 100 histological subtypes.^1^ p53 mutations, which affect approximately ~50% of sarcomas,^2, 3^ so far remain undruggable.^4^ No other common alterations have been identified across histologic subtypes.^5^ Metabolic abnormalities and dependencies are becoming recognized as opportunities for treatment,^6^ but none have yet to be identified in sarcomas as a high-frequency target. This paucity has resulted in the continued reliance on cytotoxic chemotherapies for treatment, and sarcomas remain a difficult-to-treat cancer with a very poor prognosis.^7^

Argininosuccinate synthetase 1 (ASS1) is the rate-limiting enzyme in the conversion of citrulline to arginine in the urea cycle.^8^ As arginine is a semi-essential amino acid, the loss of ASS1 makes cells dependent on extracellular sources of arginine for survival, a state referred to as arginine auxotrophy.^9^ In patients, this deficiency has grave consequences, as clearly demonstrated by the childhood disease, called citrullinemia. Citrullinemia is an autosomal recessive genetic syndrome where children are born without functional ASS1 and die from the inability to clear ammonia, because of the nonfunctional urea cycle.^10^ Cancer cells lacking ASS1 also become arginine auxotrophs, creating a targetable metabolic vulnerability across a variety of epithelial and lymphoid tumors.^11, 12, 13, 14, 15, 16, 17, 18, 19, 20, 21, 22, 23, 24^ ADI-PEG20 (pegylated arginine deiminase), a stabilized soluble form of arginine deiminase, depletes available stores of extracellular arginine and induces a metabolic stress in cells that are deficient in ASS1.^9^

The consequence of metabolic stress, such as that induced by ADI-PEG20, is usually transient autophagy, a cell survival pathway, which, in the case of arginine auxotrophs, acts primarily as a holding station to apoptosis.^21, 23, 25, 26^ In pre-clinical studies, arginine depletion by ADI-PEG20 alone is lethal in several arginine auxotrophic cancers such as prostate, breast, T-cell lymphoma and mesothelioma.^9, 13, 14, 21, 23^ Indeed, clinical trials of ADI-PEG20 are underway in hepatocellular carcinoma, acute myeloid leukemia, non-small cell lung cancer, non-Hodgkin's lymphoma, breast carcinoma, melanoma and mesothelioma. The treatment of ASS1-deficient cancers is complicated by the ability of tumors to re-express ASS1, thus there is a need to identify additional targets for synthetic lethality based on the ASS1 deficiency.

In a search for common metabolic targets in sarcomas, we profiled over 700 specimens across 45 of the most common subtypes and observed a striking absence of ASS1 in ~90% of these tumors irrespective of histology. We also demonstrate that, unlike many other tumors where ADI-PEG20 treatment alone is sufficient to induce cell death, arginine deprivation in sarcoma cell lines deficient in ASS1 produces a prolonged starvation. Moreover, by subverting this metabolic cytostasis through the addition of chloroquine, the dual metabolic stress induced apoptosis and necroptosis, an unexpected mechanism of non-apoptotic, programmed cell death. These results point to a unique synthetic lethal strategy for metabolic therapy that may have immediate clinical application across a broad swath of sarcomas.

## Results

### Prevalence and prognostic value of ASS1^low^ in sarcoma primary tumors and cell lines

**Figure 1:**
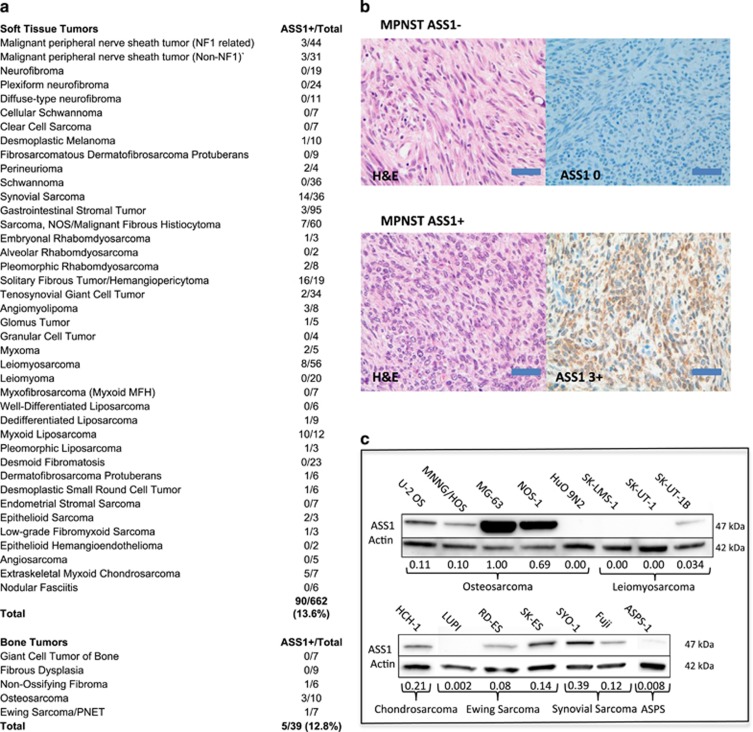
ASS1 expression level across sarcoma subtypes. (**a**) A summary of immunohistochemical detection of ASS1 in 701 primary sarcoma tumors. The tumors comprising 45 soft tissue and 5 bone sarcoma histological subtypes were examined. In all, 572/662 (86.4%) primary soft tissue and 34/39 (87.2%) bone sarcoma tumors did not exhibit strong ASS1 signal. (**b**) Typical examples of IHC. MPNST ASS1+ and ASS1- tumor samples were stained with hematoxylin and eosin (left panels) or with anti-ASS1 and counter stained with hematoxylin (right panel). Magnification x40. Scale bar=50 *μ*m. (**c**) Immunoblotting for ASS1 in a representative panel of sarcoma cells lines. In total, 13/15 (86.7%) lacked strong ASS1 expression. The osteosarcoma cell line MG-63 had the highest ASS1 expression. All ASS1 expression was normalized relative to the ASS1 high MG-63 cell line and ASS1-negative cell line SK-LMS-1. Actin serves as a loading control to ensure accurate relative ASS1 expression across cell lines

In a search for common metabolic alterations that could provide treatment opportunities for a broad range of sarcomas, we searched the literature for metabolic genes the expression of which is commonly altered in other chemorefractory tumors. Immediately, we noted that ASS1 expression was commonly lost in such tumors, including hepatocellular carcinoma, renal cell carcinoma and platinum refractory ovarian cancer.^8^ Given the chemorefractory nature of sarcoma, we decided to perform an extensive immunohistochemical analyses for ASS1 in 701 primary tumors across 45 of the most common subtypes (Figure 1a; for the full scoring of the IHC, see Supplementary Table 1). In collaboration with a sarcoma pathologist, we applied an intensity score (from 0 to 3) for ASS1 expression similar to that used for other biomarkers, such as the estrogen and androgen receptor, and also similar to that used for ASS1 in other tumors where heterogeneous expression is noted with 0 being no staining, +1 being 1–25% staining, +2 being 26–50% staining and +3 being 50% or greater staining. Positive and negative controls for ASS1 expression levels were included with each staining run, in addition vascular structures served as internal ASS1-positive staining controls. Surprisingly, a great majority of tumors (606/701, 86.4%) diagnosed as bone (34/39, 87.2%) or soft tissue sarcomas (STSs; 572/662, 86.4%) showed no signal for ASS1 expression (Figures 1a and b). We then assessed protein expression of ASS1 by immunoblotting in cancer cell lines derived from osteosarcoma (U-2 OS, MNNG/HOS, MG-63, NOS-1 and HuO 9N2), leiomyosarcoma (SK-LMS-1, SK-UT-1 and SK-UT-1B), synovial sarcoma (SYO-1 and Fuji), chondrosarcoma (HCH-1), Ewing's sarcoma (LUPI, RD-ES and SK-ES) and alveolar soft part sarcoma (ASPS-1). Consistent with the high degree of ASS1 negativity in primary sarcomas, 86.7% of the cell lines (13/15) exhibited minimal ASS1 expression (Figure 1c, see normalized expression levels). Therefore, compared with other tumors where ASS1 expression levels were observed along a continuum, results from our analyses indicate that sarcomas as a class are almost uniformly ASS1 negative or highly deficient. Our observation is in contrast to previous pilot studies in sarcoma showing that only a subset of tumors exhibited reduced ASS1 expression.^13, 14^

### ADI-PEG20 causes growth arrest in ASS1^low^ sarcoma cell lines

**Figure 2:**
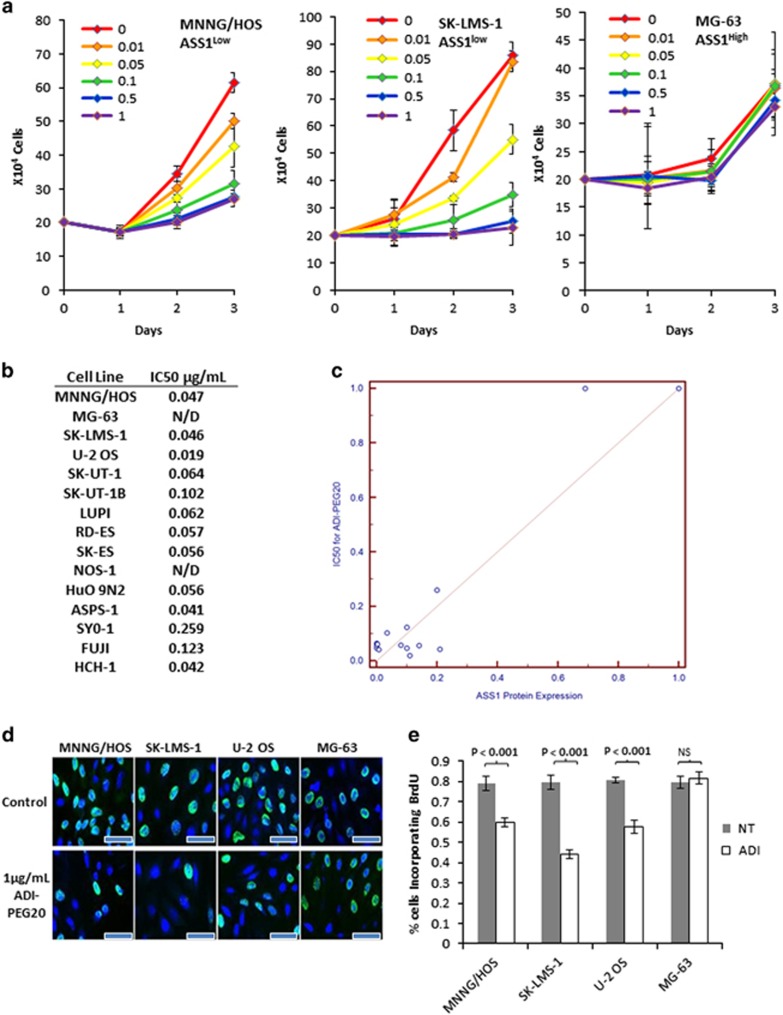
Arginine depletion causes growth arrest in ASS1^low^ cell lines. (**a**) Effects of ADI-PEG20 treatment on the growth of MNNG/HOS, SK-LMS-1 and MG-63 cell at a range of concentrations from 0 to 1 *μ*g/ml. ADI-PEG20 induced cytostasis in a dose-dependent manner in ASS1^low^ MNNG/HOS and SK-LMS-1 cells, but not in the ASS1^high^ MG-63 cells. (*N*=3). Data represented as mean±S.D. (**b**) IC50s of ADI-PEG20 in a panel of sarcoma cell lines. ND, not determined, as ADI-PEG20 had no effect on proliferation. (**c**) Correlation between ASS1 expression levels and ADI-PEG20 IC50s illustrate a higher expression level of ASS1 correlates with decreased susceptibility to growth inhibition by ADI-PEG20 treatment. (**d**) Indirect fluorescence detection of BrdU incorporation into cellular DNA. Growth inhibition as revealed by a reduction in BrdU-positive nuclei in ADI-PEG20 (at 1 *μ*g/ml) treated ASS1^low^ MNNG/HOS, SK-LMS-1 and U-2 OS cells. ASS1^high^ MG-63 cells were not affected by ADI-PEG20. These data indicate ADI-PEG20 treatment of ASS1^low^ cell lines causes cell cycle arrest. Magnification x40. Scale bar=20 *μ*m. (**e**) Quantification of BrdU-positive cells before and after treatment with ADI-PEG20 in MNNG/HOS, SK-LMS-1, U-2 OS and MG-63. (*N*=3). Data represented as mean±S.D.

The arginine auxotrophy, because of ASS1 loss in sarcomas, immediately points to the use of arginine depletion as a potentially effective therapeutic strategy. To examine the consequences of targeting this identified arginine auxotrophy in sarcoma, we measured cell numbers of the 15 sarcoma cell lines upon treating with a wide range of concentrations of ADI-PEG20 over a time course of 3 days. As predicted, cell lines such as MNNG/HOS and SK-LMS-1 with ASS1^low^ expression responded to arginine deprivation and showed reduced or static cell growth in a dose-dependent manner, but cell counts did not decrease, suggesting cytostasis rather than cell death. In contrast, ASS1^high^ cell lines MG-63 and NOS continued to proliferate, even at the highest concentration of ADI-PEG20 tested (Figures 2a and b). The growth inhibition was then used to calculate the IC50 values for each of the cell lines. Not surprisingly, ASS1 expression levels correlated with ADI-PEG20 IC50 values among our panel of sarcoma cell lines (*R*^2^=0.95, 95% CI: 0.89–0.99; *P*<0.001) (Figure 2c). There also seemed to be a threshold effect of ASS1 expression, above which cells no longer responded to ADI-PEG20 (Figure 2c).

**Figure 3:**
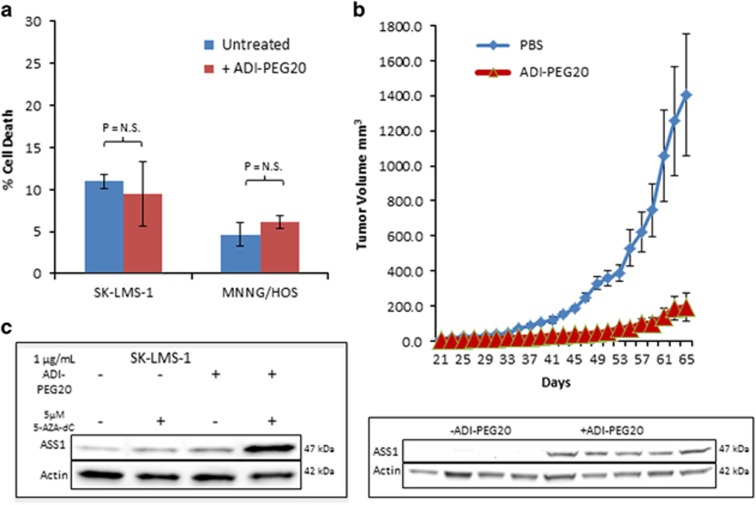
ADI-PEG20 treatment inhibits tumor growth until ASS1 re-expression confers resistance. (**a**) Flow cytometry analysis of cell death induction, as measured by percentage of cells staining positive for PI, upon treatment with ADI-PEG20. As a single agent, ADI-PEG20 does not induce cell death in these sarcoma cell lines. (*N*=3). Data represented as mean±S.D. (**b**) Tumor growth of ASS1^low^ SK-LMS-1 cells xenografted into nude mice with or without ADI-PEG20 treatment. Significant tumor growth inhibition was observed when mice were treated with ADI-PEG20 as compared with tumor growth in PBS-treated mice. Shown below is a western blot of five tumor lysates from five mice after ADI-PEG20 treatment showing re-expression of ASS1 in tumors, which had gained resistance to ADI-PEG20 mediated growth inhibition. (*N*=5 mice per arm). Data represented as mean tumor volume±S.E.M. (**c**) Re-expression of ASS1 in SK-LMS-1 cells after treatment with 1 *μ*g/ml ADI-PEG20, 5 *μ*M 5-aza-dC or both, as compared with untreated cells. After 48 h of combination treatment, ASS1 expression levels have significantly increased above wild-type conditions, as well as either drug individually. (*N*=3)

To investigate the mechanism of decreased cell growth, we performed BrdU incorporation assays and found reduced S phase entry in Ass1^low^ SK-LMS-1, MNNG/HOS and U-2 OS cells, but not in the ASS1^high^ MG-63 cells after treatment with 1 *μ*g/ml ADI-PEG20 (Figures 2d and e). Propidium iodide staining and FACS analysis revealed no evidence of cell death in any of the cell lines tested (Figure 3a). Thus, unlike other cancer cell lines deficient in ASS1 expression where treatment with ADI-PEG20 is lethal, exploitation of arginine auxotrophy by ADI-PEG20 in sarcoma cell lines results in cytostasis instead.

### Combination ADI-PEG20 and chloroquine causes synthetic lethality

Metabolic stress caused by arginine deprivation can trigger a cell survival mechanism either by: inducing an acute response in autophagy followed by the rapid re-expression of ASS1 or cell death if ASS1 is not reactivated. To determine which of these fates occurred in the case of sarcoma, we performed an *in vivo* xenograft tumor experiment using the SK-LMS-1 cell line and measured the effects of ADI-PEG20 exposure (see Materials and methods section) on tumor growth and ASS1 expression. Although ADI-PEG20 significantly retarded tumor growth, a slow and small increase in tumor size was observed over time. We harvested the tumor material after 2 months of treatment, and determined ASS1 levels in protein lysates. In each of the resistant tumors, ASS1 re-induction was clearly detected, suggesting that ADI-PEG20 treatment of sarcomas could ultimately fail because of re-expression of ASS1 (Figure 3b). Hypermethylation of the transcription promoter is a common mechanism responsible for the loss of ASS1 expression in several cancers, and previous data suggests that this is indeed the mechanism behind ASS1 silencing in myofibrosarcomas.^14, 23^ We confirmed that the low ASS1 expression in SK-LMS-1 cells was also attributable to promoter hypermethylation by exposing the cells to the demethylating agent 5-aza-2′-deoxycytidine (5-aza-dC) for 48 h. Although 5 *μ*M 5-aza-dC only slightly increased ASS1 expression, the added stress of arginine starvation with ADI-PEG20 treatment in combination with 5-aza-dC led to rapid and elevated re-expression of ASS1 (Figure 3c). We were unable to investigate whether the loss of ASS1 expression is also because of hypermethylation of the promoter in MNNG/HOS and SK-UT-1 cells, because both cell lines died after exposure to the combination of ADI-PEG20 and 5-aza-dC.

**Figure 4:**
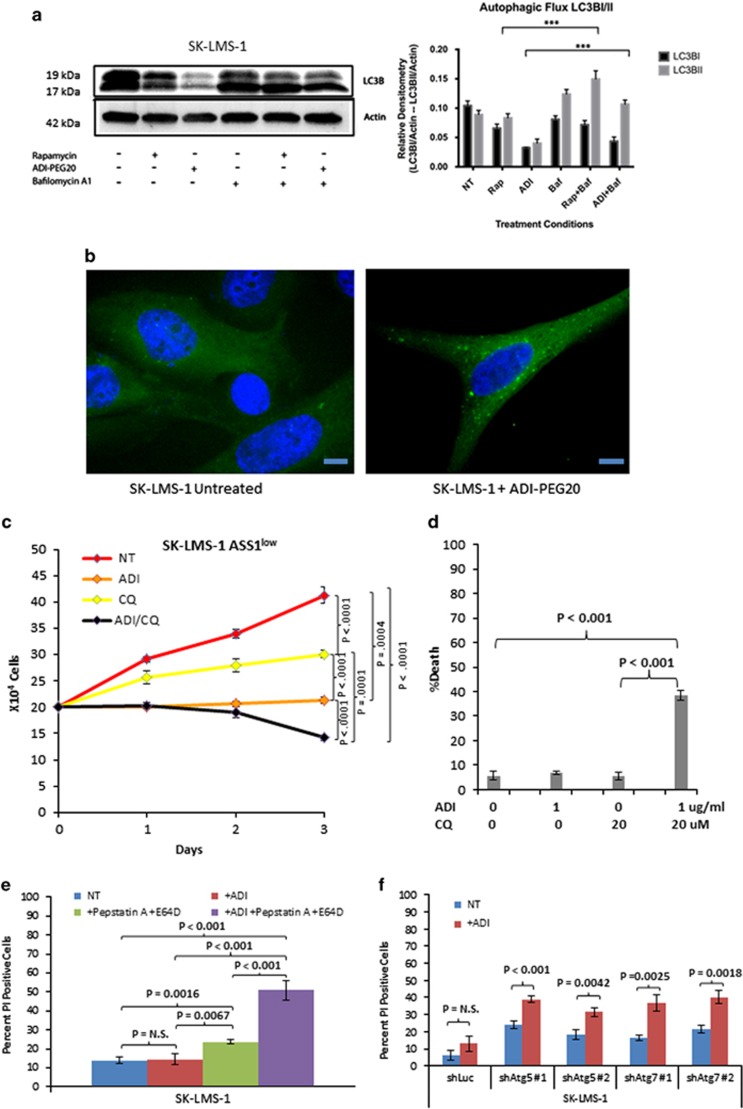
Arginine deprivation leads to a dependence on autophagy for continued cellular survival. (**a**) Cells were treated with ADI-PEG (3 days) or rapamycin (500 nM for 5 h) with or without bafilomycin A1 (100 nM) for an additional 4 h. Cell lysates were analyzed by immunoblot analysis. A representative western and bar graph, presented as means±S.D., are shown; *n*=3 (***P<0.0001). (**b**) Autophagosome formation as revealed by GFP-LC3 puncta. Lentiviral expression of GFP-LC3 was transduced into SK-LMS-1 cells that were then treated with 1 *μ*g/ml ADI-PEG20 for 3 days. A significant increase in LC3-GFP puncta demonstrates the induction of autophagy by 1 *μ*g/ml ADI-PEG20 mediated arginine deprivation in ASS1 low cells. Magnification x60. Scale bar=10 *μ*m. (**c**) SK-LMS-1 cell counts over a course of 3 days when left untreated, as well as treatment with 1 *μ*g/ml ADI-PEG20, 20 *μ*M chloroquine and both. Either agent retarded cell growth; the combination treatment significantly reduced the cell number by day 3 (*P*=0.001). (*N*=3). Data represented as mean±S.D. (**d**) Measurements of cell death upon treatment of SK-LMS-1 cells with the agents individually and together. Cell death was measured on day 3 by propidium iodide staining followed by flow cytometric analyses of treated or untreated cells. Only treatment with both agents induced significantly cell death (*P*<0.001). (*N*=3). Data represented as mean±S.D. (**e**) Measurements of cell death upon treatment of SK-LMS-1 cells with 1 *μ*g/ml ADI-PEG20, 50 *μ*M pepstatin A and 25 *μ*M E64D, individually and together. Cell death was measured on day 3 by propidium iodide staining followed by flow cytometric analyses of treated or untreated cells. Only treatment with both agents induced significantly cell death. (*N*=3). Measurements of cell death of SK-LMS-1 cells with shRNA knockdowns upon treatment with 1 *μ*g/ml ADI-PEG20 for 3 days. (*N*=3). Data represented as mean±S.D. (**f**) Measurements of cell death of SK-LMS-1 cells transduced with Luc, ATG5 or ATG7 stable knockdowns. Cell death was measured by propridium iodide staining followed by flow cytometric analyses on day 3 of ADI-PEG20 treatment

Together, these data suggest that treatment of sarcoma cells with ADI-PEG20 may induce a prolonged state of starvation before re-expression of ASS1. To test this hypothesis, we analyzed markers of autophagy by immunoblotting of whole-cell lysates over 3 days of ADI-PEG20 treatment in the ASS1^low^ sarcoma cell line SK-LMS1. The results showed that, after a treatment with 1 *μ*g/ml ADI-PEG20, there was an increase in autophagic flux, upon the addition of bafilomycin as compared with ADI-PEG20 treatment alone (Figure 4a). This may indicate the process by which sarcoma cells are able to survive in arginine starvation. We then used lentiviral expression green fluorescent protein (GFP)-LC3 in SK-LMS-1 cells and treated with ADI-PEG20 for 3 days (Figure 4b).^27^ We observed a marked increase in the number of GFP-LC3 puncta, representing autophagosomes, in ADI-PEG20-treated cells relative to untreated cells.

To verify autophagy induction, we combined ADI-PEG20 with chloroquine, which inhibits the formation of the autophagolysosome within the last steps of the autophagy process.^28,29^ SK-LMS-1 cells were treated with 1 *μ*g/ml ADI-PEG20, 20 *μ*M chloroquine or both. We found that either agent alone retarded cell growth but caused no significant increase in cell death relative to the untreated cells. However, simultaneous exposure to both agents significantly reduced the cell count (*P*=0.001) (Figure 4c) and caused extensive cell death in the SK-LMS-1 ASS1^low^ cell line (Figure 4d). Inhibition of autophagy by treatment with 50 *μ*M Pepstatin A and 25 *μ*M E64D also caused a significant increase in cell death when paired with ADI-PEG20 treatment (Figure 4e). Knockdown of two proteins essential for basal autophagy, Atg5 and Atg7, also resulted in significant increases in cell death after ADI-PEG20 treatment (Figure 4f). Thus, induction of starvation in ASS1^low^ sarcoma cell lines by arginine deprivation concomitant with chloroquine inhibition causes synthetic lethality.

### Dual therapy causes synthetic lethality via necroptosis

**Figure 5:**
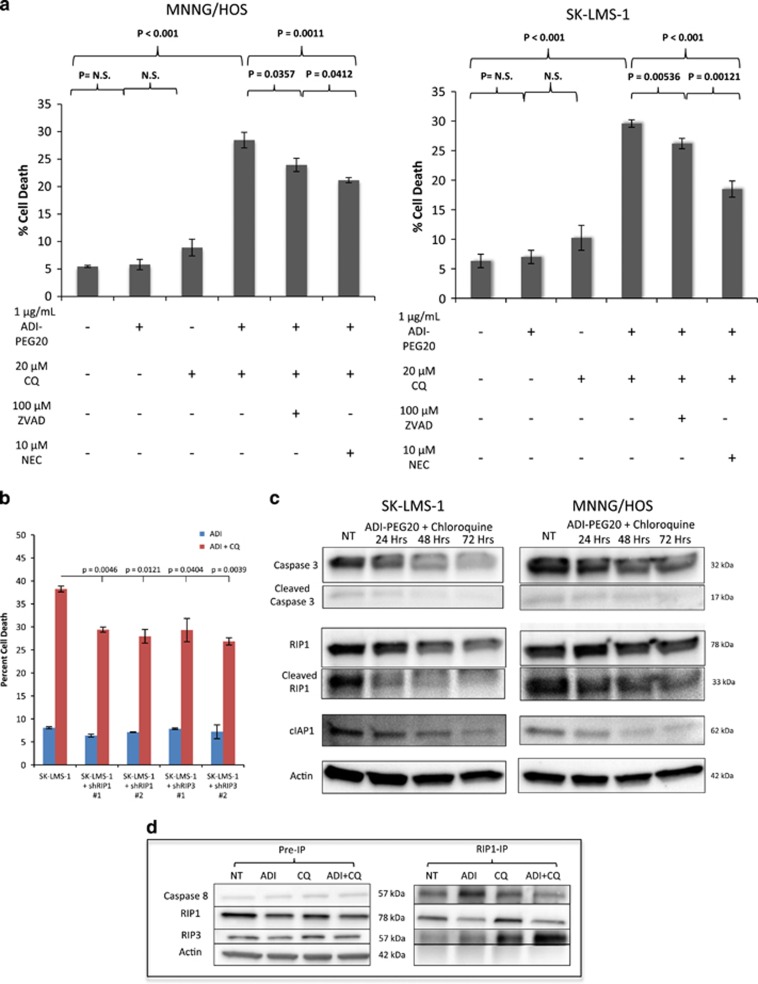
Induction of necroptosis upon simultaneous arginine deprivation and chloroquine treatment. (**a**) Cell death as measured by FACS analysis after propidium iodide uptake. MNNG/HOS (left) and SK-LMS-1 (right) cells treated with 1 *μ*g/ml ADI-PEG20, 20 *μ*M chloroquine, both, or in combination with 100 *μ*M ZVAD (an apoptosis inhibitor) or 10 *μ*M necrostatin (a necroptosis inhibitor). Protection of cell death was more effective with necrostatin, indicating cell death is occurring primarily via necroptosis. (*N*=3). Data represented as mean±S.D. (**b**) Cell death as measured by FACS analysis after propidium iodide uptake in wild type, shRIP1 or shRIP3 SK-LMS-1 cells after treatment with 1 *μ*g/ml ADI-PEG20 with or without 20 *μ*M chloroquine. RIP kinase knockdown protected from induction of cell death, indicating necroptosis induction upon dual agent treatment. Data represented as mean±S.D. (*N*=2). (**c**) Western blots of SK-LMS-1 and MNNG/HOS cells untreated, or treated with ADI-PEG20 and chloroquine for 24, 48 or 72 h. In the presence of chloroquine and ADI-PEG20, the loss of the proapoptotic cleaved caspase 3 and the anti-necroptotic cIAP1 increases the threshold for apoptosis signaling while priming cells for death by necroptosis. Decrease in levels of cleaved RIP1 further suggest necroptosis induction (**d**). RIP1 co-IP. SK-LMS-1 ASS1^low^ cells were treated with ADI-PEG20, chloroquine or both for 3 days. A significant RIP3 co-precipitation was observed upon exposure to 1 *μ*g/ml ADI-PEG20 and 20 *μ*M chloroquine, whereas caspase 8, which is negative regulator of ripoptosome formation, was reduced. Collectively, these observations are indicative of active ripoptosome formation and subsequent cell death executed preferentially by necroptosis

Next, we investigated the mechanism of cell death induced through simultaneous starvation induction with ADI-PEG20 and chloroquine in two ASS1^low^ sarcoma cell lines (SK-LMS-1 and MNNG/HOS). The cells were treated with 1 *μ*g/ml ADI-PEG20, 20 *μ*M of chloroquine or both. ZVAD (100 *μ*M), an inhibitor of apoptosis, or necrostatin (10 *μ*M), an inhibitor of necroptosis, was added individually or simultaneously in an attempt to protect against induction of cell death. Although either ZVAD or necrostatin was capable of partial protection from cell death induced by ADI-PEG20 plus chloroquine treatment, necrostatin was more effective (Figure 5a), implicating necroptosis as the dominant form of cell death. Protection from ADI-PEG20 and chloroquine-induced cell death also resulted following shRNA-mediated knockdown of receptor-interacting protein 1 (RIP1) or RIP3, further suggesting necroptosis as the mechanism of cell death upon treatment with ADI-PEG20 and chloroquine (Figure 5b).

Upon the induction of cell death pathway, a signaling cascade involving the apoptosis-associated caspase enzymes and the necroptosis-associated RIP kinases dictate the specific mechanism of cell death to be initiated. The signals for the two mechanisms of cell death processes are intertwined; with apoptosis signaling inhibiting the formation of the ipoptosome, the protein complex, which, under correct cellular contexts, can drive formation of the necroptosome.^30^ RIP1, the first essential necroptosis signaling enzyme in the ripoptosome, can be ubiquitinated and sent for proteasomal degradation by cellular inhibitor of apoptosis (cIAP1), priming cells for the induction of apoptosis upon the cellular decision to execute programmed cell death.^31, 32^ Although active caspase 8 can signal to downstream effector caspases to induce apoptosis, it can also cleave and functionally inactivate RIP1 and RIP3 kinases that are essential in signaling the initiation of necroptosis.^33, 34^ As such, when caspase 8 is functionally inactive, because of decreased protein abundance, genetic mutation or pharmacological inhibition by ZVAD, RIP kinases maintain signal transduction ability, leading to the induction of necroptosis (Supplementary Figure S1).^35, 36^ Thus, the levels of various key cell death regulatory enzymes steer the cells toward different mechanisms of programmed cell death.

Autophagy serves as a form of triage in which the cell chooses to utilize certain proteins for fuel, which it deems dispensable during starvation and we suspected that ADI-PEG20 would alter the complement of proteins in favor of necroptosis rather than apoptosis. To test this possibility, we examined by immunoblotting the effects of ADI-PEG20 and chloroquine on several proteins involved in either apoptosis or necroptosis pathways. Immunoblots of lysates of cells treated with ADI-PEG20 alone showed that cleaved poly ADP ribose polymerase (PARP) and BCL2 remained constant (Supplementary Figure S2). However, there was a decrease in the abundance of proteins involved in apoptosis, such as cIAP1, caspase 3, and caspase 8. The loss of cIAP1 primes cells for ripoptosome formation.^31^ The decrease in caspase 8, a negative regulator of RIP1 activation, also preferentially led the cells toward necroptosis (Supplementary Figure S2).^34^ In addition, the decrease in cleaved caspase 3 increased the activation threshold needed to trigger apoptosis. Analysis of protein levels at 24, 48 and 72 h of ADI-PEG20 and chloroquine treatment showed a significant decrease in cIAP1, full length and cleaved caspase 3, and full length and cleaved RIP1 (Figure 5c). Decreases in cleaved caspase 3 and cleaved RIP1 further indicate induction of necroptosis. The different protein patterns detected between the MNNG/HOS and SK-LMS-1 cell lines when treated with chloroquine alone likely reflect variant levels of basal autophagy activation. Interestingly, the protein levels seen in cells treated with the combination of ADI-PEG20 and chloroquine on day 3 were virtually identical to those present in cells treated with ADI-PEG20 alone (Supplementary Figure S2). The protein levels reflect contents of cells that have survived the combination drug treatment and were in the autophagic state. Alternatively, the literature suggested that chloroquine may have chemosensitization properites independent of autophagy, which may play into the effect we observe.^37^

In order to substantiate ripoptosome formation, we performed immunoprecipitation of RIP1 from SK-LMS-1 cells to determine RIP1-associated proteins after the different treatment conditions (Figure 5d). Importantly, there was a significant increase in the abundance of associated RIP3, along with a decrease in caspase 8, indicating the formation of the active ripoptosome, the complex that initiates the cellular process of necroptosis. Taken together, these observations demonstrate necroptosis as the primary mechanism of cell death (Supplementary Figure S1), similar to previous studies showing induction of necroptosis resulting from simultaneous inhibition of mTOR and autophagy.^38^

### Therapeutic efficacy of combination treatment

**Figure 6:**
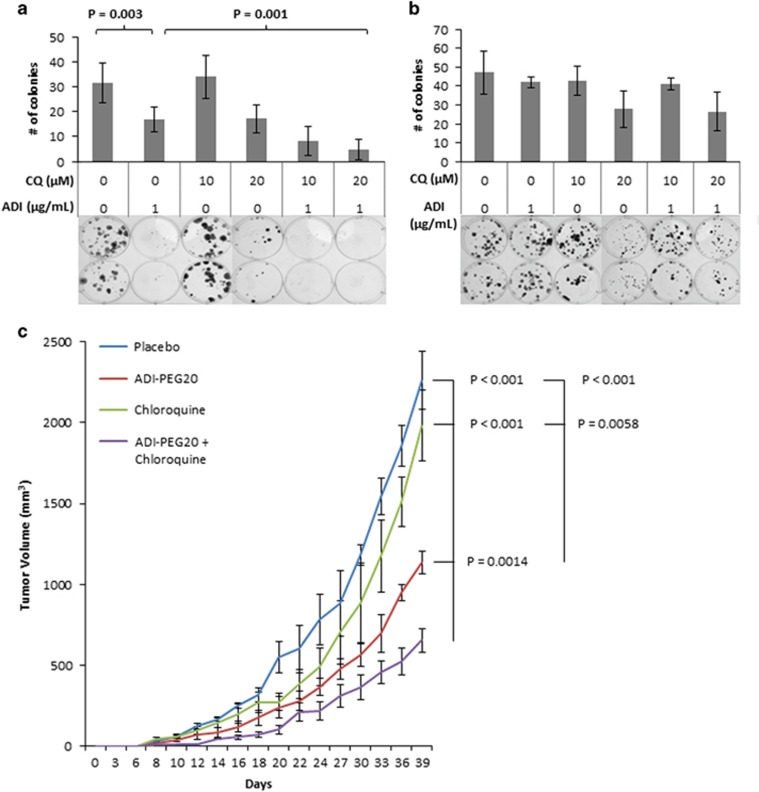
*In vivo* efficacy of synthetic lethal therapeutic targeting strategy. (**a** and **b**) Colony formation upon treatment with 1 *μ*g/ml ADI-PEG20, 10 *μ*M or 20 *μ*M chloroquine or both. (**a**) Combination treatment of SK-LMS-1 ASS1^low^ cells significantly inhibited long-term colony formation and colony size, especially when the chloroquine was used at 20 *μ*M (*P*=0.001). (*N*=3). Data represented as mean±S.D. (**b**) The effects on MG-63 ASS1^high^ cells were moderate and were attributable to chloroquine alone, whereas ADI-PEG20 had little effect. (*N*=3). Data represented as mean±S.D. (**c**) Tumor volumes of ASS1^low^ MNNG/HOS osteosarcoma cells xenografted into nude mice. Combination ADI-PEG20 and chloroquine treatment significantly inhibited tumor growth *versus* PBS, ADI-PEG20, or chloroquine treatment alone. Data represented as mean tumor volume±S.E.M. (*N*=5 mice per arm)

We next investigated the potential utility of combined ADI-PEG20 and chloroquine as a therapeutic strategy. First, we performed classic colony-forming assays whereby cells were plated at a low density, exposed to either chloroquine, ADI-PEG20 or both for 1 week, and then allowed to grow in the absence of the compounds for another week (Figure 6a). In the ASS1-deficient cell line SK-LMS-1, treatment with ADI-PEG20 reduced colony number by approximately 30% while simultaneously causing very small colonies, because of the cytostatic effect of ADI-PEG20. The effects of chloroquine treatment on colony formation varied depending on the working concentration used. Although 10 *μ*M chloroquine inhibits autophagy in low serum conditions, 20 *μ*M is required for inhibition of autophagy in the presence of serum.^39^ Consistent with these results, treatment with 10 *μ*M of chloroquine had no effect on either ASS1-positive or -negative cells when cultured in our standard serum-containing media. There was a minor but significant effect on colony number and size when cells were treated with 20 *μ*M chloroquine, regardless of the ASS1 status (Figures 6a and b). In contrast, when cells were treated with ADI-PEG20, the presence of 10 *μ*M chloroquine further reduced the size and number of colonies in the ASS1^low^ cell line SK-LMS-1. The effects were even more pronounced when chloroquine was used at 20 *μ*M (Figure 6a). The reduction in colony number and size in the ASS1^high^ cell line MG-63 treated with both agents was moderate and primarily attributable to chloroquine alone (Figure 6b).

The above observations suggested that both ADI-PEG20 and chloroquine would be needed to produce an antitumor effect *in vivo*. Therefore, we performed a proof-of-principle experiment by using subcutaneous xenografts of osteosarcoma-derived ASS1^low^ MNNG/HOS cells (Figure 6c) into the flanks of immunodeficient nude mice. This cell line was chosen for its higher basal ASS1 level of expression when compared with SK-LMS-1 or SK-UT-1, allowing for a quicker escape from ADI-PEG20 treatment compared with the SK-LMS-1 xenografts utilized in Figure 3a. This model would inform on improved treatment regimen to control tumors in future pre-clinical studies. After injection of the MNNG/HOS osteosarcoma cell line, mice were treated with PBS control, ADI-PEG20, chloroquine or a combination of the two for 24 days. We found that, at doses effective for other tumors,^39^ single-agent chloroquine had no effect on sarcoma tumor growth, whereas the single-agent treatment with ADI-PEG20 delayed and reduced tumor growth. However, the dual-agent therapy was able to decrease tumor volumes (*P*<0.001) significantly (Figure 6c). These *in vivo* data confirm the efficacy of combining ADI-PEG20 with chloroquine. Additional studies will be necessary to optimize the treatment regimen to improve efficacy.

## Discussion

The last 20 years have ushered in explosive progress in the treatment of most solid tumors with the notable exception of sarcoma, outside gastrointestinal stromal tumors. A major reason for this lack of progress is the heterogeneity and presumed lack of common lesions that can be exploited for therapeutic purposes in this disease. We now show that sarcoma as a disease is characterized by arginine auxotrophy owing to the loss of ASS1 expression. This realization led us to test strategies exploiting this vulnerability. We show that a combination of different metabolic stresses can exert a significant effect on tumor growth via necroptosis activation, in addition to slightly lower levels of apoptosis activation, without the need for cytotoxic conventional chemotherapy. These results open up an enormous avenue for basic and clinical investigation in treating sarcomas.

Sarcomas consist of 70 different tumor subtypes that can be divided into bone and STSs depending on the cell of origin.^1^ Genetically, sarcomas frequently have mutations in p53 (50%), Rb (to a lesser extent) and occasional PI3K mutations (<1%), but none of the mutations identified in sarcoma (other than gastrointestinal stromal tumor (GIST) with cKIT and PDGF mutations) is either druggable or is high-frequency treatment targets.^2, 3, 40^ Sarcomas often have complex cytogenetics or can be translocation driven, and among the latter are hybrid transcription factors like EWS-Fli in Ewing's sarcoma, SYT-SSX in synovial sarcoma, Pax3/7:FKHD in rhabdomyosarcoma, ASPL:TFE3 in alveolar soft part sarcoma and FUS:CHOP is myxoid liposarcoma.^5, 41^ However, these translocations are only represented in a very small minority of tumors and by and large do not provide treatment opportunities. In this context, our finding of a near universal ASS1 loss provides the first druggable genetic alteration in sarcoma that encompasses all histologies.

Based on our data, sarcomas now can be defined as a cancer, which is auxotrophic for arginine, but the consequences of this auxotrophy are different from most other tumor types. Most commonly, single-agent treatment of ASS1-deficient cancer cells with ADI-PEG20 induces apoptosis. Representative tumors that exhibit this biology are T-lymphoblastic leukemia, melanoma and small cell lung cancer.^18, 23, 42^ ADI-PEG20 treatment of prostate cancer is unique in that arginine starvation in these tumor cells causes chromatophagy, a form of autophagy in which cells start incorporating chromatin into autophagosomes, followed by cell death.^42, 43^ In contrast, sarcoma cells are able to use sustained autophagy until they reprogram and re-express ASS1, a change that renders single-agent ADI-PEG20 treatment ineffective. However, this starvation primes the cells for induction of necroptosis because of the loss of anti-ripoptosome regulatory proteins, as well as caspase 3, a rate-limiting protein that initiates apoptosis. These altered death protein compositions are likely the consequence of autophagy, and dictate that necroptosis will be the preferential mechanism of cell death upon chloroquine treatment. Therefore, by arresting the completion of autophagy with chloroquine, the cell can no longer engage this survival mechanism, and necroptotic death preferentially ensues. These data are the first demonstration of a metabolic therapy leading to necroptosis.

In sum, we have identified a translatable, well-tolerated, metabolic approach to treat an essentially untreatable disease. The full extent of the metabolic changes induced by ADI-PEG20-dependent starvation in these mesenchymal arginine auxotrophs needs to be further characterized to exploit fully and to refine this therapeutic opportunity. Evolving proteomics approaches should enable a detailed analysis of the cellular protein compositions that are altered during arginine starvation. This information will yield further insight into building multi-agent metabolic treatments for sarcoma that can avoid the need for chemotherapy, or potentially increase the efficacy of traditional chemotherapy. Moreover, this approach can serve as a model for evolving therapies for other difficult-to-treat cancers.

## Materials and Methods

### Primary tumor immunohistochemistry

In all, 701 tumors from patients diagnosed with sarcoma, including 45 histologic soft tissue subtypes, were examined for ASS1 protein expression by immunohistochemistry. After initial excision, tumor samples were fixed in 10% neutral buffered formalin and embedded in paraffin. Blocks were sectioned to 5*μ*m for immunohistochemical detection of ASS1 protein expression levels. The anti-ASS1 antibody was a gift from Polaris Pharmaceuticals, Inc. (San Diego, CA, USA) Glass sections were digitally scanned using the Aperio ScanScope CS System (Aperio, Vista, CA, USA) and analyzed.

### Cell lines and reagents

All cell lines were obtained from the American Type Culture Collection (Chicago, IL, USA), with the following exceptions: HCH-1 was from the laboratory of Dr. Linda Sandell at Washington University in St. Louis, MO, USA, and ASPS-1 was from the laboratory of Dr. Robert Shoemaker at the National Cancer Institute (Bethesda, MD, USA). SK-LMS-1, MNNG/HOS, MG-63, SK-UT-1 and SK-UT-1B cells were maintained in minimum essential media (Invitrogen, Waltham, MA, USA). Fuji, RD-ES, LUPI, NOS-1, HuO 9N2, HCH-1 and SK-ES cells were maintained in RPMI (Invitrogen). U-2 OS cells were maintained in McCoy's medium (Invitrogen). ASPS-1 cells were maintained in DMEM/F12 (Invitrogen). 293 T and SYO-1 cells were maintained in DMEM (Invitrogen). All media were supplemented with 10% fetal bovine serum (Invitrogen) and penicillin–streptomycin (Invitrogen). Chloroquine (used at 10 and 20 *μ*M), 5-aza-dC (5 *μ*M), necrostatin (10 *μ*M) and ZVAD-FMK (100 *μ*M) were obtained from Sigma Aldrich (St. Louis, MO, USA). Pepstatin A (used at 50 *μ*M) and E64D (used at 25 *μ*M) were obtained from Enzo Life Sciences (Farmingdale, NY, USA). ADI-PEG20 (used at 0.01, 0.05, 0.1, 0.5 and 1 *μ*g/ml for dose-response curve, and at 1 *μ*g/ml for all other *in vitro* experiments) was obtained from Polaris Pharmaceuticals, Inc. Lentiviral GFP-LC3 was a generous gift from Conrad Weihl (Washington University in St. Louis).^27^

### Viability assays

Cell death was quantified by annexin-V (BioVision, Milpitas, CA, USA) or propidium iodide (Sigma, St. Louis, MO, USA) staining, followed by flow cytometric analyses. Flow cytometry was performed using a FACSCalibur (BD Biosciences, San Jose, CA, USA). Data were analyzed using FloJo software (Ashland, OR, USA). *P*-values for statistical analyses were obtained using Student's *t*-test.

### Xenograft mouse model

All animal experiments were approved by the IACUC at Washington University in St. Louis. One million ASS1^low^ MNNG/HOS cell (Figure 5c) or SK-LMS-1 cells (Figure 3b) were injected subcutaneously in the rear flank fat pad of immune-deficient nude mice. Treatment was initiated on day 1. Mice were treated with PBS, ADI-PEG20, chloroquine or the combination of ADI-PEG20 and chloroquine. Chloroquine was given subcutaneously at 60 mg/kg daily.^39^ In all, 320 IU/m^2^ (29.1 mg/m^2^) ADI-PEG20 was intramuscularly injected biweekly. Starting on day 6, tumors were measured every other day and tumor volumes were calculated by 1/2 x (length x width^2^). Mice were killed when tumors in the control arm reached a maximum size of 2000 mm^3^.

### Immunoblotting and co-immunoprecipitation (co-IP)

Antibodies used for immunoblots were as follows: anti-ASS1 (Polaris, San Diego, CA, USA), anti-LC3 (Sigma), anti-p62 (Sigma), anti-RIP1 (Cell Signaling), anti-caspase 8 (Cell Signaling), anti-BCL-2 (Cell Signaling), anti-cIAP1 (Cell Signaling), anti-cleaved PARP (Cell Signaling), anti-caspase 3 (Imgenex, San Diego, CA, USA), anti-RIP3 (Abcam, Cambridge, MA, USA), anti-Atg5 (Santa Cruz, Dallas, TX, USA), anti-Atg7 (Microbiology Laboratories, Woburn, MA, USA), and anti-actin (Sigma). Cells were lysed in RIPA buffer and protein concentrations were determined by BCA kit (Pierce, Waltham, MA, USA). In all, 25–40 *μ*g of proteins was resolved by NuPAGE (Invitrogen) and transferred onto PVDF membranes (Immobilon-P, Millipore, Darmstadt, Germany). Antibody detection was accomplished using enhanced chemiluminescence (Western Lightning, PerkinElmer, Melville, NY, USA).

For co-IP, SK-LMS-1 cells were treated with PBS, 1 *μ*g/ml ADI-PEG20, 20 *μ*M chloroquine, or both for 3 days. Following treatment, cells were lysed in 0.2% NP-40 buffer and protein concentration was determined by BCA kit (Pierce). Lysates were incubated with anti-RIP1 (Cell Signaling) and protein A/G beads (Pierce). The immunoprecipitates were subsequently immunoblotted with anti-RIP3 (Abcam), anti-RIP1 (Cell Signaling), anti-caspase 8 (Cell Signaling) and anti-actin (Sigma) as described above.

## Abbreviations

ASS1: argininosuccinate synthetase 1
ADI-PEG20: pegylated arginine deiminase
GFP: green fluorescent protein
RIP: receptor-interacting protein
cIAP1: cellular inhibitor of apoptosis
PARP: poly ADP ribose polymerase
STS: soft tissue sarcoma
GIST: gastrointestinal stromal tumor
