# Supplementary figures and images for: A metabolic synthetic lethal strategy with arginine deprivation and chloroquine leads to cell death in ASS1-deficient sarcomas
Source: Cell Death Dis. 2016 Oct 13;7(10):e2406–. doi: 10.1038/cddis.2016.232 (PMC5133958; doi:10.1038/cddis.2016.232)

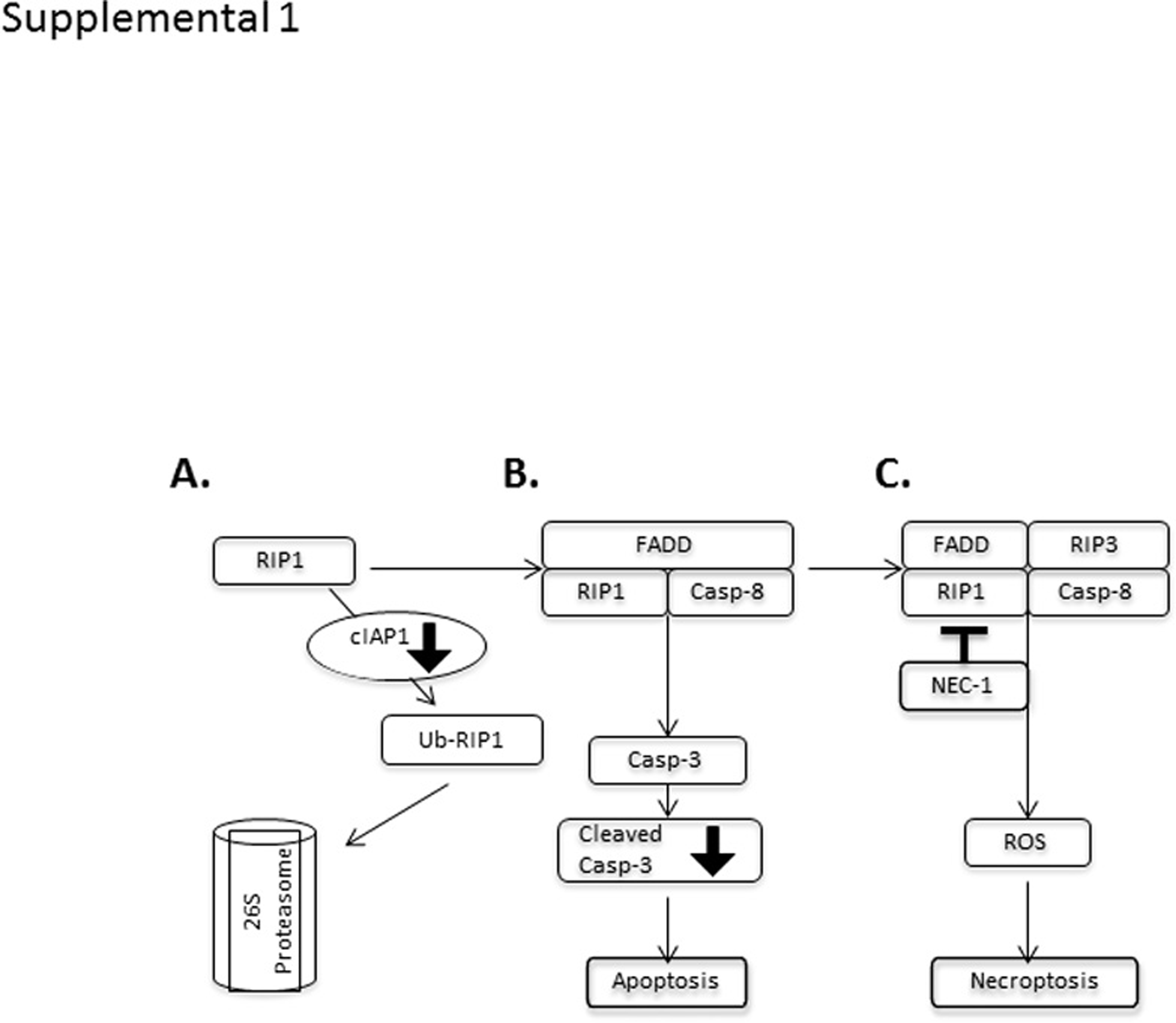

Supplement: Supplementary Figure S1 [file cddis2016232x1.tif]

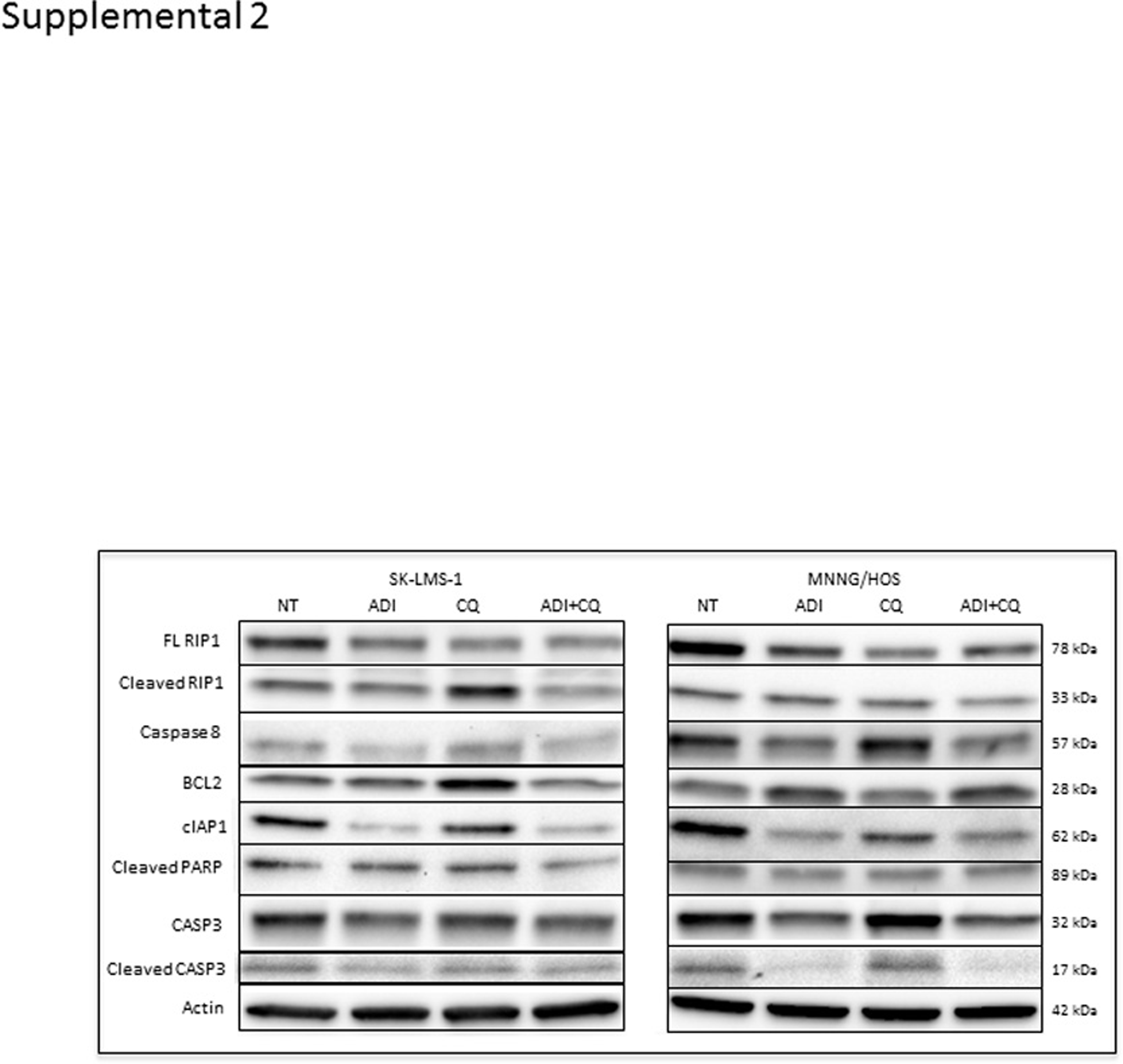

Supplement: Supplementary Figure S2 [file cddis2016232x2.tif]
