# Supplementary material for: A metabolic synthetic lethal strategy with arginine deprivation and chloroquine leads to cell death in ASS1-deficient sarcomas
Source: Cell Death Dis. 2016 Oct 13;7(10):e2406–. doi: 10.1038/cddis.2016.232 (PMC5133958; doi:10.1038/cddis.2016.232)
Supplement: Supplementary Table Legend [file cddis2016232x4.docx]

Supplementary Table 1 Ledged:

Table of ASS1 expression in 662 soft tissue and 39 bone sarcomas divided by individual histology as determined by IHC. Samples were scored on a scale of 0-+3, with 0 being no expression, +1 scoring being 1-25% of cells positive, +2 scoring being 26-50% of cells positive, and +3 scoring being greater than 50% of cells positive.
